# Perturbation of Host Cell Cytoskeleton by Cranberry Proanthocyanidins and Their Effect on Enteric Infections

**DOI:** 10.1371/journal.pone.0027267

**Published:** 2011-11-04

**Authors:** Kevin Harmidy, Nathalie Tufenkji, Samantha Gruenheid

**Affiliations:** 1 Department of Chemical Engineering, McGill University, Montreal, Quebec, Canada; 2 Complex Traits Group and Department of Microbiology and Immunology, McGill University, Montreal, Quebec, Canada; Charité-University Medicine Berlin, Germany

## Abstract

Cranberry-derived compounds, including a fraction known as proanthocyanidins (PACs) exhibit anti-microbial, anti-infective, and anti-adhesive properties against a number of disease-causing organisms. In this study, the effect of cranberry proanthocyanidins (CPACs) on the infection of epithelial cells by two enteric bacterial pathogens, enteropathogenic *Escherichia coli* (EPEC) and *Salmonella* Typhimurium was investigated. Immunofluorescence data showed that actin pedestal formation, required for infection by enteropathogenic *Escherichia coli* (EPEC), was disrupted in the presence of CPACs. In addition, invasion of HeLa cells by *Salmonella* Typhimurium was significantly reduced, as verified by gentamicin protection assay and immunofluorescence. CPACs had no effect on bacterial growth, nor any detectable effect on the production of bacterial effector proteins of the type III secretion system. Furthermore, CPACs did not affect the viability of host cells. Interestingly, we found that CPACs had a potent and dose-dependent effect on the host cell cytoskeleton that was evident even in uninfected cells. CPACs inhibited the phagocytosis of inert particles by a macrophage cell line, providing further evidence that actin-mediated host cell functions are disrupted in the presence of cranberry CPACs. Thus, although CPAC treatment inhibited *Salmonella* invasion and EPEC pedestal formation, our results suggest that this is likely primarily because of the perturbation of the host cell cytoskeleton by CPACs rather than an effect on bacterial virulence itself. These findings have significant implications for the interpretation of experiments on the effects of CPACs on bacteria-host cell interactions.

## Introduction

The consumption of cranberry has been linked with the prevention and treatment of urinary tract infections for over 100 years. However, a mechanistic understanding of the way in which cranberry materials prevent bacterial infection is still lacking. Some studies suggest that a specific fraction of the cranberry known as proanthocyanidins (PACs) is responsible for its anti-infective properties [1], [2], [3], [4].

PACs are part of a group of chemicals known as flavonoids and can be found in many other fruits, seeds, leaves and nuts. In addition to PAC, flavonoid compounds include anthocyanins, flavonols and catechins and are often collectively referred to as “extracts” [5]. At certain concentrations, cranberry flavonoids have been attributed antiviral properties [6], [7] as well as antimicrobial properties against many important human pathogens, including *Escherichia coli*, *Helicobacter pylori*, *Listeria monocytogenes*, *Porphyromonas gingivalis*, *Salmonella* Typhimurium, and *Staphylococcus aureus* [8], [9], [10], [11], [12], [13]. In addition to these observed antiviral and antibacterial properties, cranberry flavonoids also exhibit effects directly on mammalian cells. Specifically, they have been associated with the induction of apoptosis of adenocarcinoma cells [14], [15], [16], [17], have exhibited anti-inflammatory activity [15], [18] and have been shown to act as a cardiovascular protector [19], [20]. Increasingly, PACs are believed to be the subgroup of flavonoids responsible for these effects.

Cranberry PACs (CPACs) have been linked with a reduction in bacterial adhesion onto biological [2], [3], [21], [22], [23], [24] and non-biological [25], [26] surfaces. Proposed mechanisms of action include CPACs' potent antioxidant capacity [27], [28], metal chelation [29], [30], blocking motility [31], [32] or by simple steric interference between bacteria and a target surface [25]. Few studies, however, have examined the impact of CPACs directly on host cells, during their interaction with pathogenic bacteria.

CPACs are high molecular weight compounds made up of flavan-3-ol monomers [2]. While still open for debate, it is believed that lower-order polymers are absorbed into the bloodstream subsequent to ingestion, leaving higher-order polymers intact in the gastrointestinal (GI) tract [5], [33]. If higher-order CPACs are not metabolized, it becomes of interest to study the effect of CPACs on GI health. Therefore, since CPACs may be present in the GI tract, and have the potential to act on GI pathogens directly and to affect their adhesion to surfaces, we decided to characterize the interaction of gut pathogens with host cells in the presence of CPAC.

Two important gut pathogens were chosen as models for enteric infection. Enteropathogenic *Escherichia coli* (EPEC) is a major cause of infantile diarrhoea [34] while *Salmonella* Typhimurium is one of the key strains causing salmonellosis [35]. To date, this is the first study to examine the role of CPACs in EPEC and *Salmonella* infection. Our results demonstrate that CPACs protect epithelial cells from infection by these two important gut pathogens. Furthermore, we provide evidence that the protection observed is not due to an antimicrobial or anti-infective effect of CPACs on the bacteria, but rather results from alterations of the host cell cytoskeleton in the presence of CPACs. These findings have important implications for studies on the effect of CPACs and related compounds on host-pathogen interactions.

## Results

**Figure 1:**
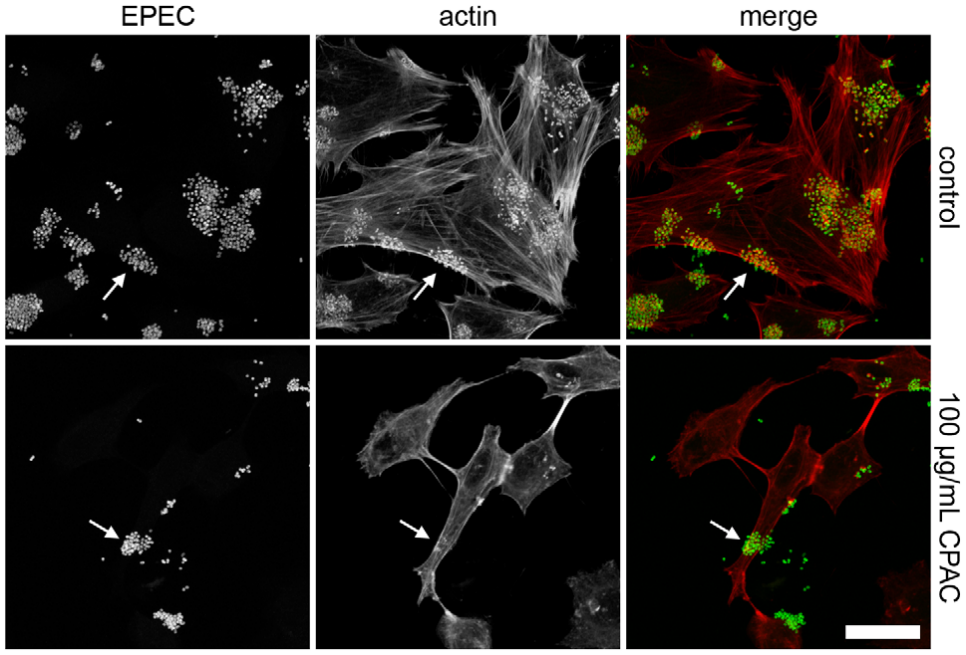
EPEC pedestal formation in the presence of CPAC. HeLa cells infected for 6 hours with EPEC. Left panel is EPEC-GFP (green), actin staining in the centre panel (red) and a colour overlay on the right. Control experiment (top panels) demonstrates the presence of actin pedestals beneath bacteria (top arrows). In the presence of 100 µg/mL CPAC (bottom panels), localization of actin beneath EPEC bacteria is disrupted (bottom arrows). An overall change in the number of adhered bacteria as well as a change in HeLa cell morphology is also observed. Experiments were carried out in triplicate and a single representative image set was used for each condition. Scale bar indicates 25 µm.

A fundamental characteristic of EPEC infection of host cells is the formation of actin pedestal structures located directly beneath adherent bacteria [36], [37]. Pedestal formation requires the type III secretion system mediated translocation of a bacterial protein, Tir, into the host cell membrane. Tir has an intracellular domain that mediates the polymerization of host cell actin at the site of bacterial attachment [38]. To assess the effect of CPACs on the interaction of EPEC with host cells, we examined pedestal formation in the presence and absence of a purified CPAC preparation. In cells infected with EPEC but untreated with CPACs, robust actin polymerization was evident at the site of bacterial attachment (Figure 1, white arrows, top panels). However, localized actin polymerization was greatly diminished in the presence of CPACs (Figure 1, white arrows, bottom panels). Although CPACs had no effect on bacterial growth at all concentrations tested (see below), the number of adherent EPEC bacteria was also significantly reduced in the CPAC treated sample (Figure 1, left panels). Analysis of CLSM images of HeLa cells revealed that 9.5%±0.3 of the cell surface area was covered with EPEC under control conditions, but only 2.7%±0.8 of the cell surface area was covered with EPEC when the cells had been exposed to CPACs. CPACs also exerted similar effects during infection of HeLa cells with RDEC-1, a rabbit pathogen closely related to EPEC (Figure S1).

**Figure 2:**
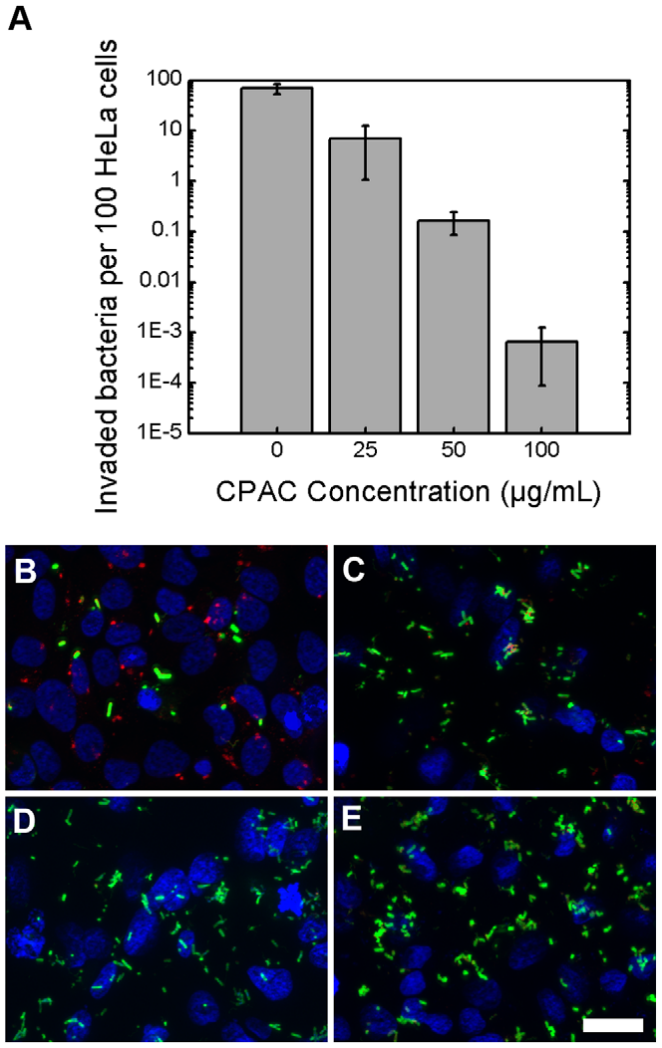
Invasion of HeLa cells by *Salmonella* Typhimurium. HeLa cells infected with *S.* Typhimurium for 40 minutes were enumerated (panel *a*) or visualized by immunofluorescence (IF, panels *b–e*). IF staining was performed such that internalized bacteria is represented in red and external bacteria is represented in green. *S.* Typhimurium readily invades cells when no CPAC is present at a frequency of about 1 bacterium per HeLa cell (see panel *a* and *b*). In the presence of 25 µg/mL CPAC (panel *c*), this frequency is diminished by roughly 90%. Panels *d* and *e* represent a CPAC concentration of 50 and 100 µg/mL respectively and near negligible amounts of invasion was observed. Scale bar indicates 25 µm. Error bars on graph represent one standard deviation. Experiments were carried out separately, in triplicate, and a single representative sample was chosen for each concentration.

Whereas EPEC is an intestinal pathogen that is able to infect from an extracellular position, *Salmonella* Typhimurium invades epithelial cells and replicates intracellularly [39]. To assess the effect of CPACs on the interaction of an invasive intestinal pathogen with epithelial cells, we performed two independent *S.* Typhimurium invasion assays in the presence and absence of CPACs, using the *S.* Typhimurium Keller strain. A gentamicin protection assay was used to enumerate internalized bacteria (Figure 2*a*), while immunofluorescence was used to visualize the cells and bacteria (Figure *2b–e*). The gentamicin protection assay revealed a potent and dose-dependent inhibition of *Salmonella* invasion by CPACs with a 10-fold reduction at the lowest concentration tested (25 µg/mL) and over a 100 000-fold reduction in invaded bacteria at 100 µg/mL. This effect was confirmed in the immunofluorescence images. In the latter assay, bacteria that have invaded the HeLa cell are shown in red, whereas *S.* Typhimurium cells that remain outside the host cell are stained green. Figure 2*b* shows that a large number of bacteria are able to invade the HeLa cells under control conditions. In contrast, Figure 2*e* reveals that nearly no bacteria in the field have been internalized in the presence of CPACs. These results show that CPACs strongly inhibit *S.* Typhimurium invasion into HeLa cells (Figure 2). CPACs also potently decreased the host cell invasion of two additional *Salmonella* Typhimurium strains, as shown by the gentamicin protection assay (Figure S2).

**Figure 3:**
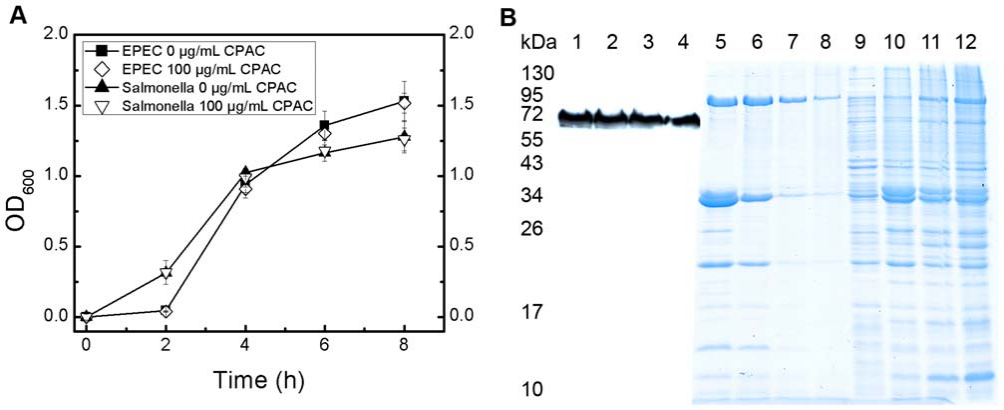
Bacterial growth and secretion of EPEC in the presence of CPAC. Growth of EPEC and *S.* Typhimurium in the presence of 0 and 100 µg/mL CPAC was examined over 8 hours. CPAC had no effect on the growth of both bacterial strains (Figure 3*a*). EPEC total proteins were resolved by 12% SDS-PAGE in the presence of increasing amounts of CPAC before probing for Tir protein on Western Blot (wells 1–4); 0, 25, 50, 100 µg/mL CPAC respectively). EPEC secreted proteins after 30 min incubation with 0, 25, 50, 100 µg/mL CPAC (supernatant, wells 5–8 respectively; pellet, wells 9–12 respectively). CPAC had no effect on the production of effector proteins, but readily precipitates proteins (Figure 3*b*).

After observing these changes in infection levels, we examined whether CPAC treatment was affecting the growth of EPEC and Salmonella. Growth curves were performed over 8 hours on both bacterial species in the absence and presence of increasing amounts of CPACs. CPACs had no effect on the growth of either EPEC or *Salmonella* at CPAC concentrations of 100 µg/mL (Figure 3*a*). We next assessed whether CPACs were acting as inhibitors of bacterial type III secretion. Type III secretion systems are important virulence determinants of a number of gram-negative pathogens, including EPEC and *S.* Typhimurium. They act by translocating bacterial effector proteins into host cells to subvert host cell functions to favour bacterial replication or survival. Type III secretion is required for both EPEC pedestal formation and *S.* Typhimurium invasion [38], [39]. Furthermore, it has previously been reported that bacterial type III secretion is inhibited by flavonoids from citrus fruit [40], [41]. Assays have been developed to assess type III effector secretion into growth medium in the absence of host cells. In these assays, bacteria are grown in secretion-inducing medium and effector-containing supernatants are collected following removal of the bacterial cells by centrifugation. Initially, we observed an absence of type III secreted effector proteins in the supernatants in the presence of CPACs (data not shown). However, further experimentation revealed that addition of CPACs to secreted protein fractions prepared in the absence of CPACs also lead to their precipitation and removal from the supernatant upon centrifugation (Figure 3*b*). This is consistent with a previous report that CPACs can precipitate proteins [42]. Indeed, we found that the secreted proteins were present in the pellets of the CPAC-treated samples after centrifugation (Figure 3*b*). Thus, precipitation of proteins by CPACs makes assessment of the effect of CPACs on type III secretion unfeasible. Due to this technical limitation, we assessed the production (rather than secretion) of type III-secreted effector proteins by EPEC, using Western blotting of total bacterial extracts, and found that they had no effect (Figure 3*b*, wells 1–4). Based on these results we hypothesized that the anti-infective properties of CPACs noted here may be linked to effects on the host cell.

**Figure 4:**
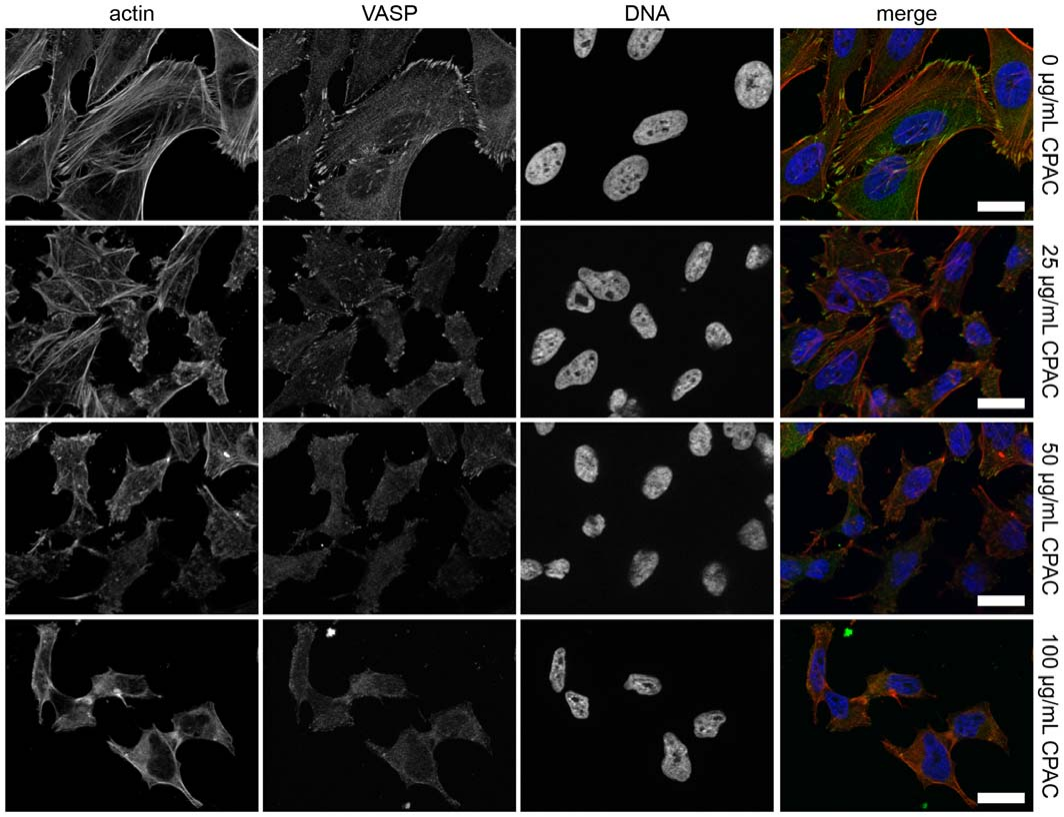
Assessment of the effect of CPAC on the host cell cytoskeleton. HeLa cells were exposed to three concentrations of CPAC for 4 hours before using immunofluorescence to visualize cell structure. Cells were stained for actin (red, left), for vasodilator-stimulated phosphoprotein (VASP, green, second column), and DAPI (blue, third column). A colour composite image is shown in the right column. Control experiments (top panels) demonstrate normal actin arrangement as well as localization of VASP protein to the focal adhesion points. In a dose-dependent manner, actin arrangement is disrupted, in parallel with the delocalization of the VASP protein from the focal adhesion points as established by the second (25 µg/mL CPAC), third (50 µg/mL CPAC) and fourth (100 µg/mL CPAC) rows. The effect on the actin cytoskeleton is similar to the effect seen in Figure 1. Scale bars indicate 25 µm.

**Figure 5:**
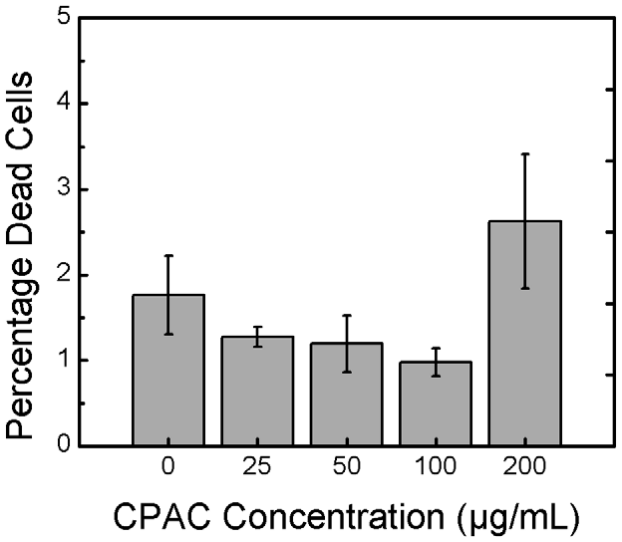
Viability of HeLa cells. HeLa cells were treated with varying concentration of CPAC for 4 hours before determining viability using a viability/cytotoxicity kit and fluorescence imaging. CPAC had no effect on the viability of epithelial cells after 4 hours and at the concentrations tested. Error bars represent one standard deviation from the analysis of a minimum of six photos per concentration.

A common element between EPEC pedestal formation and *Salmonella* Typhimurium invasion into host cells is the requirement for specific bacterially-induced rearrangements of the host cell actin cytoskeleton. We noted that the actin staining in the micrographs of EPEC infected cells appeared very different in the CPAC treated and untreated cells (Figure 1, middle panels). To test whether CPACs could affect the actin cytoskeleton in the absence of infection, we treated uninfected HeLa cells with increasing concentrations of CPACs for a duration of 4 hours and then stained the cells for polymerized actin and for several cytoskeleton-associated proteins. The images show that CPAC treatment alone leads to a perturbation of the cytoskeleton in a dose-dependent manner (Figure 4). Changes in polymerized actin, cell shape and size, and localization of focal adhesion proteins (VASP) were evident even with the lowest concentration of CPACs examined (25 µg/mL). At 50 µg/mL CPACs, most of the VASP protein no longer localized at the focal adhesions and the shape of the cell was markedly changed. At 100 µg/mL, CPACs had the most dramatic effect on cell shape, size, and protein localization. Two other actin cytoskeleton-associated proteins tested (α-actinin and paxillin) also demonstrated the same type of delocalization similar to that noted for VASP in Figure 4 (data not shown). To verify whether the notable reorganization of the cytoskeleton led to damage of the HeLa cells, we conducted a viability assay in the presence and absence of CPACs. HeLa cells were treated with CPACs for 4 hours and viability was determined using a commercial staining kit which differentiates dead cells according to their permeability to ethidium homodimer-1. No significant change in viability was observed for CPACs concentrations ranging from 0–100 µg/mL (Figure 5). We observed a slight increase in the number of dead cells in the presence of 200 µg/mL CPAC, though this result was not found to be statistically significant.

**Figure 6:**
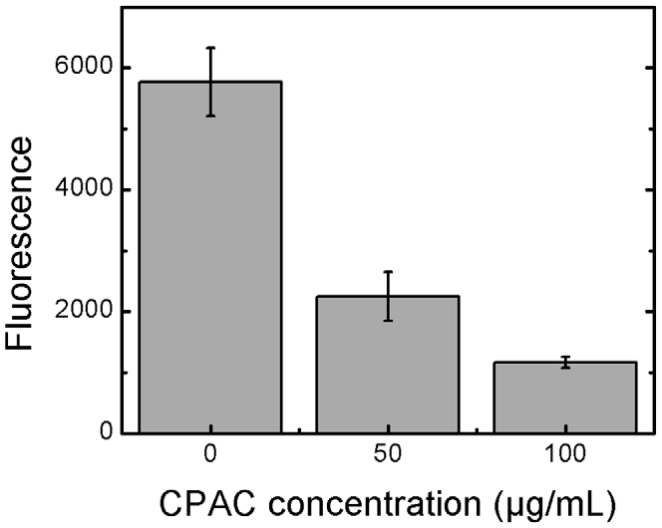
Phagocytosis of fluorescent BioParticles. Particles conjugated to a pH-sensitive fluorophore will fluoresce subsequent to internalization by a macrophage cell. In the presence of 50 µg/mL CPAC, fluorescence is reduced three-fold, indicating that the phagocytosis of these particles was reduced. In the presence of 100 µg/mL, this was reduced six-fold compared to the control. Error bars represent one standard deviation from one experiment performed in triplicate.

Though HeLa cell viability was unaffected, we hypothesized that the cytoskeletal changes induced by CPACs would lead to a disruption of normal cytoskeletal function. To test this hypothesis in a non-infection model, and to verify whether the effects of CPACs were generalizable to multiple cell types, we studied the phagocytosis of inert model particles by J774 murine macrophage cells. Like EPEC pedestal formation and *Salmonella* invasion, phagocytosis requires extensive remodelling of the actin cytoskeleton. To assess phagocytosis, we used BioParticles which fluoresce only when exposed to the acidic pH of the phagosomal environment. This can be quantified in a plate reader. Under control conditions (no CPACs), BioParticles were readily taken up by macrophage cells and fluoresced brightly. However, in the presence of CPACs, uptake of BioParticles by murine macrophages was reduced significantly and in a dose-dependent manner (Figure 6). To ensure that the decrease in fluorescence correlated with decreased phagocytosis of the particles, samples were also examined by fluorescent microscopy and decreased phagocytosis of particles in the presence of CPACs was observed (data not shown).

## Discussion

In this work, we studied the effect of CPACs on infection of cells with two important gastrointestinal pathogens, EPEC and *Salmonella* Typhimurium. CPACs inhibited EPEC attachment to cells (Figure 1) and prevented actin pedestal formation by EPEC, a process thought to be of critical importance for the establishment of a productive EPEC infection. CPACs also markedly reduced infection of epithelial cells by *Salmonella* Typhimurium (Figure 2). Notably, *Salmonella* was incapable of invading host cells even at relatively low concentrations of CPACs (10-fold reduction at 25 µg/mL). This suggests that CPACs can possibly prevent enteric infections in the body, though further *in vivo* studies regarding the bioavailability of CPACs are required.

While still open for debate, it is believed that lower-order CPAC polymers are absorbed into the bloodstream subsequent to ingestion, leaving higher-order polymers intact in the GI tract where they could potentially act on GI bacteria [5], [33]. To date, a clear link between diets high in PAC-containing foods and reduced GI infection has not been established, but the current findings indicate that further study may be warranted.

EPEC pedestal formation and *Salmonella* host cell invasion require viable bacteria and the translocation of bacterial effector proteins into host cells via the bacterial type III secretion system. An attempt was made to determine the effect of CPACs directly on bacteria. Though other studies have demonstrated the use of cranberry compounds as an antimicrobial agent [8], [9], [10], [11], [12], [13], there is no evidence to support this in the current study (Figure 3*a*). This may be due to the CPAC concentrations tested here. In addition, although other groups have reported that other fruit flavonoids can inhibit production of type III secreted proteins [40], [41], we found that CPACs had no direct effect on the expression of EPEC effector proteins under the conditions we employed (Figure 3*b*). Although not tested, it is likely that the expression of *Salmonella* effector proteins was also unaffected by CPACs because of the similarity of the two type III secretion systems. Though no measurable effect was observed on bacterial growth or effector protein levels, we cannot exclude the possibility that CPACs did have other effects on the bacterial cells that were not assessed here. However, our finding that CPACs had a dramatic and dose-dependent effect on the host epithelial cell cytoskeleton (Figure 4), offers a probable explanation for one of the key CPAC anti-infective mechanisms. In order to infect the host cell, EPEC and *Salmonella* rely on the hijacking of cellular machinery, and in particular, they manipulate the host actin cytoskeleton [38], [39]. We hypothesized that the actin cytoskeleton alterations induced by CPACs rendered the cytoskeleton resistant to the bacterially-induced remodelling normally directed by type III-translocated effector proteins. We predicted that if this were true, CPAC treatment would also render the actin cytoskeleton resistant to non-bacterially-induced actin remodelling. To test this, we examined the phagocytosis of non-living particles in the presence or absence of CPACs. Professional phagocytic cells such as macrophages engulf foreign particles through the process of phagocytosis. The process requires extensive remodelling of the actin cytoskeleton and is sensitive to inhibitors of actin polymerization. The uptake of these model particles was dramatically reduced in the presence of CPACs (Figure 6).

One caveat of the work presented here is that these studies were performed in HeLa cells and a mouse macrophage cell line. While HeLa cells are a commonly used and widely accepted model cell line to assess both EPEC pedestal formation and *Salmonella* invasion, they do not accurately model all aspects of the polarized epithelia of the GI tract. It will be of interest to extend our findings in polarized intestinal epithelial cell lines and in *in vivo* infection models. Notably, we have observed striking changes in the actin cytoskeleton of the polarized intestinal epithelial cell line Caco-2 upon CPACs treatment (data not shown), and current efforts in our labs include further characterizing these effects.

The mechanism by which CPACs induce perturbation of the actin cytoskeleton is not known, but ongoing work in our laboratory aims to address this question. Other flavonoids have been shown to affect the actin cytoskeleton, but the mechanisms by which they do so are not well characterized [43], [44]. Indeed, PACs from many other fruits, berries, barks and seeds have been attributed related properties as was recently reviewed by de la Iglesia et al. [27]. Many studies, however, have pointed to the fact that cranberries contain PACs with A-type linkages that may have different properties than the more common B-type PAC [27], [45]. Therefore, it is unclear whether grape seed PACs, for example, will have the same effect as CPACs, as in the current study. As purification techniques improve and these types of compounds are isolated further into dimers, trimers etc. extensive testing should be carried out and the results can be compared across PACs from several species. In addition to this, the bioavailability of these groups of compounds needs to be studied further, as very little is known about the fate of these compounds after ingestion [33]. To date, few studies have looked at the effects of PACs *in vivo*, and whether or not they are able to reach their target tissue intact.

The effect of CPACs on GI health was systematically evaluated using different *in vitro* models. CPACs were found to reduce infectivity of two intestinal pathogens, without affecting the viability of either the bacteria or the host. In addition, CPACs cause a rearrangement of host cell cytoskeleton, and as a result, are able to block invasion in a non-specific manner. Because CPACs are able to act non-specifically and do not affect viability, these cranberry extracts may offer a potential alternative to antibiotics. A key advantage over conventional antibiotics is that CPACs do not pose the risk of antibiotic resistance and could potentially cost much less than current options. Future studies should evaluate the use of CPACs *in vivo*, and their effects on the infection of common intestinal pathogens.

## Materials and Methods

### Cranberry proanthocyanidin

Dry PAC extract from the American cranberry species *Vaccinium macrocarpon* was isolated and purified as described previously [2]. Dry CPAC powder was solubilized in deionized (DI) water to obtain a CPAC stock solution of 1.5 mg/mL which was subsequently filtered with a 0.20 µm syringe filter and stored at 4°C in the dark. Prior to experimentation, CPAC was diluted to the desired concentration from the stock solution.

### Strains, plasmids and growth media

The streptomycin-resistant (Sm^r^) EPEC O127:H6 strain E2348/69 [46] was used for the majority of experimentation. EPEC-GFP was made by transforming EPEC with the kanamycin resistant plasmid pAT113-GFP [47]. Rabbit diarrheagenic *E. coli* strain 1b (RDEC-1b) was provided by Dr. Josée Harel (Université de Montréal). Three *Salmonella enterica* serovar Typhimurium (*S.* Typhimurium) isolates were also used. Specifically, strains Keller [48], SL1344 [49] and 14028S (ATCC).

A single colony from an agar plate was used to inoculate 5 mL of media in a glass culture tube, and incubated overnight at 37°C and shaking at 220 rpm. For *Escherichia coli*, lysogeny broth (LB, 5 g yeast extract (EMD Chemicals), 10 g sodium chloride (Fisher) and 10 g tryptone (EMD) per litre of DI) was used as medium, while trypticase soy broth (TSB, Becton-Dickinson) was used for *Salmonella*. Agar plates were made by adding 15 g agar (Bioshop) per litre media.

Bacterial growth curves were obtained by diluting an overnight culture 1∶100 into 5 mL fresh media containing 0 or 100 µg/mL CPAC and incubating at 37°C and 220 rpm. A sample was removed every two hours and measurements were obtained by reading the optical density at 600 nm.

### Cell culture

All cells were maintained at 37°C and 5% CO_2_ in a humidified incubator in high glucose Dulbecco's Modified Eagle Medium (DMEM, Invitrogen) with 10% heat-inactivated foetal bovine serum (iFBS, Thermo Scientific). HeLa cells (ATCC CCL-2) were passaged 1∶10 once 60–90% confluency was reached using 0.25% Trypsin with EDTA (Invitrogen) and incubating for 3 minutes. J774 murine macrophage (ATCC TIB-67) cells were harvested and passaged at 1∶10 as needed. Macrophage cells were passaged by replacing used media, scraping cells into fresh media, centrifuging for 3 minutes at 500 *g* and adding resuspended cells to a new flask. Passage numbers between 5 and 25 were used for experiments involving the different cells.

### Pedestal assay

Assessment of pedestal formation was performed as previously described [50]. Briefly, 24 hours prior to infection, HeLa cells were plated at a density of 2×10^5^ cells per well in a 24-well plate (Costar) containing sterile round glass coverslips and incubated at 37°C and 5% CO_2_. An overnight culture of EPEC-GFP was diluted to a multiplicity of infection (MOI) of 50 into serum-free DMEM containing no CPAC (control) or 100 µg/mL CPAC and incubated for 1 hour at 37°C and 5% CO_2_. Media in the 24-well plates was then replaced with the prewarmed media containing bacteria, and plates were incubated for 6 hours at 37°C and 5% CO_2_. Cells were then washed three times with phosphate buffered saline (PBS) containing 100 mM calcium and 100 mM magnesium to remove non-adhered free-floating bacteria. Cells were fixed with 2.5% paraformaldehyde (PFA, Canemco) for 15 minutes at room temperature.

Following fixation, cells were permeabilized and blocked with 0.1% Triton X-100 (BioShop)/10% normal goat serum (NGS, Invitrogen) in PBS for 30 minutes at room temperature. Actin was stained with Alexa 568-conjugated phalloidin (Invitrogen) diluted 1∶100 into 0.1% Triton X-100/10% NGS in PBS. Following this, cells were washed three times with PBS and once with DI before being mounted using ProLong Gold Anti-fade Reagent (Invitrogen) and curing overnight.

Images were acquired using a Carl Zeiss Laser Scanning Microscope (LSM) 5 Exciter with Argon laser at 488 nm (green) and Helium-Neon laser at 543 nm (red). Emission light was split to allow Channel 1 (red) to receive wavelengths above 560 nm and Channel 2 to receive wavelengths between 505–530 nm. Of each sample, three images were taken, containing a Z-stack of 25 slices each for a thickness of about 7 µm. Images were Z-projected using ImageJ and representative images were selected for Figure 1. For the purposes of bacterial enumeration, the green image was analyzed further. Threshold values were set to represent bacteria in black and background in white and software measurement tools calculated the percentage of bacterial coverage.

### Gentamycin protection assay

Using 24-well plates, HeLa cells were set out at a density of 2×10^5^ cells/mL in 500 µl DMEM+10% iFCS. *S.* Typhimurium cells were grown overnight and then diluted 1∶15 in fresh TSB media containing 0, 25, 50 or 100 µg/mL CPAC. After 1.5 hours of incubation at 37°C and 220 rpm, the bacterial suspension was adjusted to an optical density of 0.9 (600 nm) and further diluted 1∶100 into fresh DMEM containing the target concentration of CPAC. This medium was used to replace the HeLa cell medium and plates were centrifuged at 500 *g* for 5 minutes to promote bacterial contact with host cells. Following a 40 minute incubation period at 37°C and 5% CO_2_, wells were washed with PBS, and DMEM containing 100 µg/mL gentamycin (Invitrogen) was added for 1 hour. Wells were washed again with PBS and cells were lysed with 1% Triton X-100. Wells were serially diluted, plated onto agar plates, and enumerated manually.

For imaging, HeLa cells were plated in 24-well dishes containing sterile glass coverslips as described above. Following infection, cells were washed and fixed as described above. Inside-outside staining was performed by using unconjugated rabbit anti-*Salmonella* as primary antibody (Cedar Lane, diluted 1∶100) and Alexa-Fluor 488 or 568 (diluted 1∶100) goat anti-rabbit IgG as secondary antibodies. Cells were treated with primary antibody for 1 hour, washed three times with PBS and then treated with an Alexa 488-conjugated secondary antibody for 30 minutes. Following three more washes with PBS, cells were permeabilized with 0.1% Triton X-100/10% NGS in PBS for 15 minutes and staining was repeated but using an Alexa 568-conjugated secondary antibody. Cells were treated with DAPI (4′,6-diamidino-2-phenylindole, Sigma), diluted 1∶100 for 5 minutes and mounted using ProLong Gold.

Imaging was performed on a Zeiss Axiovert fluorescent microscope equipped with an apotome. Twelve Z-slices totalling 3 µm thick were obtained for each image, and a minimum of 5 images were taken per slide. Z-projections were prepared using ImageJ. The red image was subtracted from the green image using ImageJ and the subsequent RGB image is obtained. External bacteria are thus shown in green whereas internal bacteria are shown in red. Representative images were taken from one of four experimental repeats.

### Secretion assay

EPEC from an overnight culture was diluted 1∶50 into serum-free DMEM containing 0, 25, 50 and 100 µg/mL CPAC and incubated at 37°C and 5% CO_2_. After 4 hours, secreted proteins were separated from whole cells by 5 minutes centrifugation at 16 000×g. Proteins pellets were collected in 160 µL sodium dodecyl sulfate polyacrylamide gel electrophoresis (SDS-PAGE) loading buffer before resolution by 12% SDS-PAGE for 1.5 hours and 100 V. A western blot was used to visualize the effect of CPAC on type-III secreted protein Tir. Briefly, proteins were transferred onto PVDF membrane and blocked with 5% (w/v) milk in TBST overnight at 4°C. Primary antibody, mouse anti-Tir was diluted 1∶5000 in TBST+5% milk and incubated for 2 hours before washing and using anti-mouse conjugated HRP diluted 1∶5000 for an additional 2 hours. Western blot was exposed to film for 10 seconds before developing.

Additionally, EPEC secreted proteins were collected and incubated with 0, 25, 50, 100 µg/mL CPAC for 30 min and 220 rpm. Samples were centrifuged for 5 min at 16 000×g. Proteins in pellet were collected with SDS-loading buffer. Proteins in supernatant were precipitated by 10% (v/v) trichloroacetic acid for 1 hour at 4°C and collected with SDS-loading buffer after centrifugation at 16 000×g for 20 minutes and 4°C before running on 12% SDS-PAGE.

### Cytoskeletal protein visualization

HeLa cells were set out on glass coverslips in 24-well plates as described above. After 24 hours of incubation, media was replaced to contain 0, 25, 50 or 100 µg/mL CPAC in serum-free DMEM and further incubated for 4 hours at 37°C and 5% CO_2_. A variety of cytoskeletal antibodies were used to examine the effect of CPAC. Mouse anti-vasodilator-simulated phosphoprotein (VASP), mouse anti-α-actinin and mouse anti-paxillin were diluted 1∶100 and used as primary antibodies for immunofluorescence. After fixing, blocking and permeabilizing (as described above), cells were incubated for 1 hour with primary antibody. Cells were washed and incubated with Alexa-Fluor 488 conjugated goat anti-mouse IgG, Alexa 568-conjugated phalloidin and DAPI diluted 1∶100 for 30 minutes at room temperature. Samples were washed and mounted as described above. Images were acquired using a Zeiss Axiovert fluorescence microscope with apotome. Five images were taken of each sample at 63× magnification. Representative images of VASP protein were included in the figure. Similar effects were observed for the other cytoskeletal proteins examined (data not shown).

### Mammalian cell viability/cytotoxicity

HeLa cells were set out on glass coverslips in 24-well plates as described above. After 24 hours of incubation, media was replaced to contain 0, 25, 50, 100 or 200 µg/mL CPAC in serum-free DMEM and further incubated for 4 hours at 37°C and 5% CO_2_. Cells were washed three times and viability was assessed using a Live/Dead Viability/Cytotoxicity kit for mammalian cells (Invitrogen). A solution of 2 µM calcein AM and 4 µM ethidium homodimer-1 was used for 45 minutes to stain live cells green and dead cells red. Imaging was performed directly after staining using a Zeiss Axiovert fluorescence microscope at 4× magnification. A minimum of six images were acquired at each concentration of CPAC. Red images were analyzed to obtain the percent coverage of dead cells using ImageJ.

### BioParticle assay

Murine macrophage cells were set out at a density of 1×10^6^ cells/mL in clear-bottomed black 96-well plates (Costar) and allowed to settle for 1.5 hours at 37°C and 5% CO_2_. pHrodo *E. coli* BioParticles were purchased from Invitrogen and prepared according to manufacturer recommendations. CPAC was added to BioParticles to 0, 50 and 100 µg/mL. Cell culture media was replaced with BioParticle suspension containing the various concentrations of CPAC and incubated for 3 hours at 37°C. BioParticles are conjugated to a fluorogenic dye that dramatically increases in fluorescence as the surrounding pH becomes more acidic; namely, when the BioParticle becomes internalized by macrophage cells. Wells were read using fluorescent plate reader (Synergy MX) at an excitation and emission of 560/585 mn. Fluorescence values from control wells containing BioParticles but no cells were subtracted from the values of those containing cells.

## Supporting Information

Figure S1 RDEC-1b pedestal formation in the presence of CPAC. HeLa cells were infected for 6 hours with Rabbit Diarrheagenic *E. coli* strain 1 (RDEC-1b; kindly provided by Dr. Josée Harel, Universite de Montreal) in the presence or absence of 100 µg/mL CPACs, then fixed and stained as described in the materials and methods section. Left panels are RDEC stained with rabbit-anti-*E. coli* antibody (Thermo Scientific), centre panels are Alexa-568 phalloidin staining and colour overlays are shown on the right (E. coli in green and phalloidin staining in red). Control experiment (top panels) demonstrates the presence of striking actin recruitment beneath adherent bacteria (e.g. near arrowhead). In the presence of 100 µg/mL CPACs (bottom panels), localization of actin beneath RDEC bacteria was greatly diminished (e.g. near arrowhead) and the number of adherent bacteria was reduced.
(JPG)

Figure S2 Invasion of HeLa cells by *Salmonella* Typhimurium. *S.* Typhimurium strains SL1344 and 14028S were grown overnight and seeded 1∶15 in fresh TSB media containing 0, 25, 50 or 100 µg/mL CPAC. After 1.5 hours of incubation at 37°C and 220 rpm, the bacterial suspension was adjusted to an optical density of 0.9 (600 nm) and further diluted 1∶100 into fresh serum-free DMEM containing the target concentration of CPAC. This medium was used to replace medium in wells, and plates were centrifuged at 500 g for 5 minutes. HeLa cells were infected with both S. Typhimurium strains for 40 minutes, and internalized bacteria were enumerated manually by direct plate counting. *S.* Typhimurium readily invades cells when no CPAC is present at a frequency of about 1 bacterium per 10 HeLa cells. In the presence of 25 µg/mL CPAC, this frequency is diminished roughly 100%. Near negligible amounts of bacteria were found to have invaded at CPAC concentrations of 50 and 100 µg/mL. Error bars on graph represent one standard deviation.
(TIF)
