# Supplementary figures and images for: Perturbation of Host Cell Cytoskeleton by Cranberry Proanthocyanidins and Their Effect on Enteric Infections
Source: PLoS One. 2011 Nov 4;6(11):e27267. doi: 10.1371/journal.pone.0027267 (PMC3208605; doi:10.1371/journal.pone.0027267)

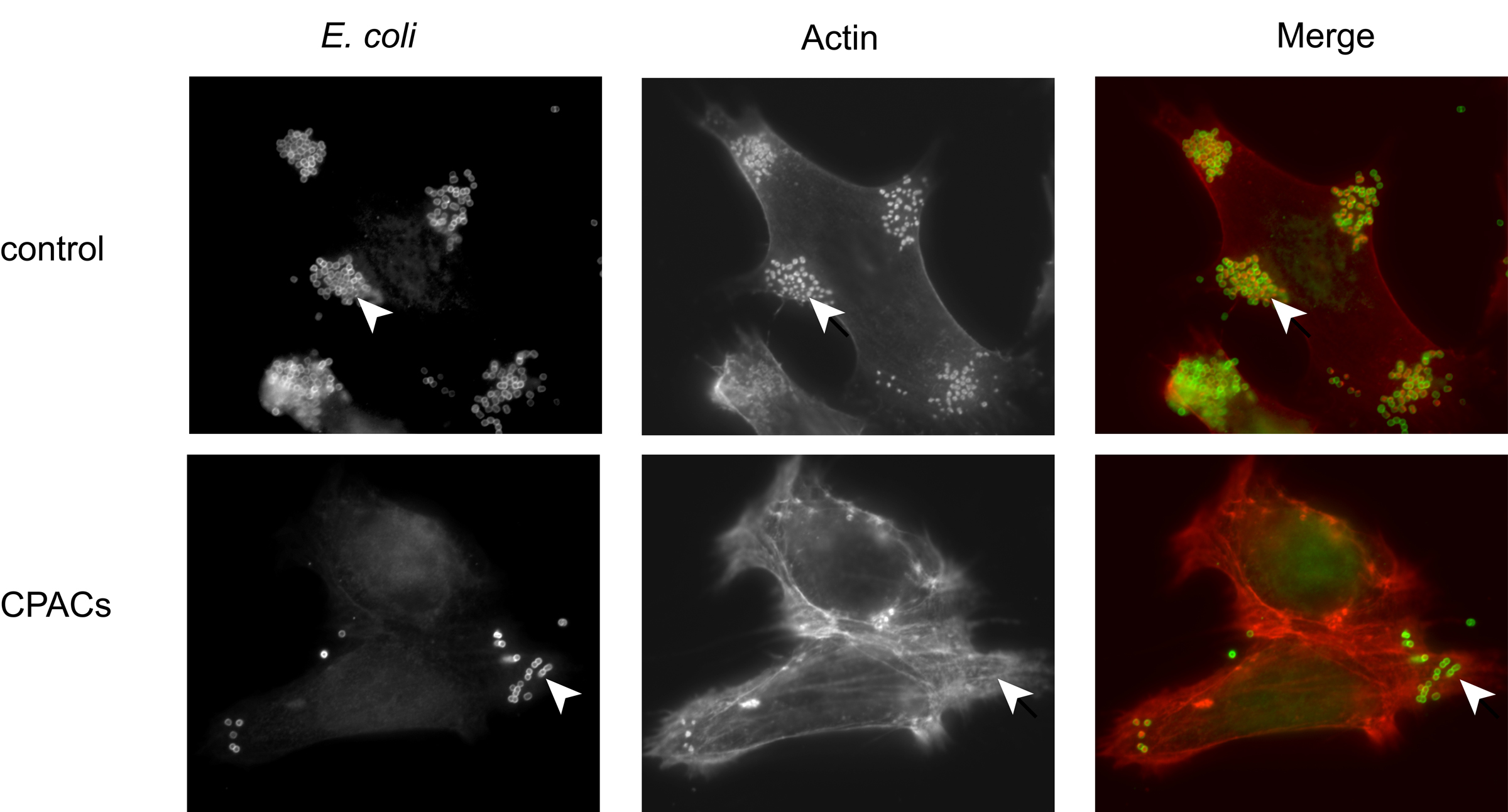

Supplement: Figure S1 — RDEC-1b pedestal formation in the presence of CPAC. HeLa cells were infected for 6 hours with Rabbit Diarrheagenic E. coli strain 1 (RDEC-1b; kindly provided by Dr. Josée Harel, Universite de Montreal) in the presence or absence of 100 µg/mL CPACs, then fixed and stained as described in the materials and methods section. Left panels are RDEC stained with rabbit-anti-E. coli antibody (Thermo Scientific), centre panels are Alexa-568 phalloidin staining and colour overlays are shown on the right (E. coli in green and phalloidin staining in red). Control experiment (top panels) demonstrates the presence of striking actin recruitment beneath adherent bacteria (e.g. near arrowhead). In the presence of 100 µg/mL CPACs (bottom panels), localization of actin beneath RDEC bacteria was greatly diminished (e.g. near arrowhead) and the number of adherent bacteria was reduced. (JPG) [file pone.0027267.s001.jpg]
